# Integrated network analysis and effective tools in plant systems biology

**DOI:** 10.3389/fpls.2014.00598

**Published:** 2014-11-04

**Authors:** Atsushi Fukushima, Shigehiko Kanaya, Kozo Nishida

**Affiliations:** ^1^RIKEN Center for Sustainable Resource ScienceTsurumi, Yokohama, Japan; ^2^Japan Science and Technology Agency, National Bioscience Database CenterTokyo, Japan; ^3^Graduate School of Information Science, Nara Institute of Science and TechnologyNara, Japan; ^4^Laboratory for Biochemical Simulation, RIKEN Quantitative Biology CenterOsaka, Japan

**Keywords:** network visualization, pathway analysis, flux balance analysis, genome-scale metabolic reconstruction, plant metabolism

## Abstract

One of the ultimate goals in plant systems biology is to elucidate the genotype-phenotype relationship in plant cellular systems. Integrated network analysis that combines omics data with mathematical models has received particular attention. Here we focus on the latest cutting-edge computational advances that facilitate their combination. We highlight (1) network visualization tools, (2) pathway analyses, (3) genome-scale metabolic reconstruction, and (4) the integration of high-throughput experimental data and mathematical models. Multi-omics data that contain the genome, transcriptome, proteome, and metabolome and mathematical models are expected to integrate and expand our knowledge of complex plant metabolisms.

## Introduction

Plants are a paramount source of food, energy, and valuable compounds. The developing field of plant systems biology has provided outstanding insights into how these products are synthesized; its ultimate goal is an understanding of the genotype-phenotype relationship in cellular systems (Kell, 2002; Benfey and Mitchell-Olds, 2008; Weckwerth, 2011). Recent technical advances in high-throughput sequencing and various analytical instruments have made it possible to comprehensively measure and analyze genes, transcripts, proteins, and metabolites (Fukushima et al., 2009; Lei et al., 2011; Lucas et al., 2011; Stitt, 2013). These omics technologies are not only platforms that monitor the cellular inventory, but they also provide the opportunity to evaluate cellular behaviors from a multi-level perspective and enhance our understanding of plant systems (Krouk et al., 2010; Saito and Matsuda, 2010; Dhondt et al., 2013).

Major effective and efficient approaches to analyze omics data are network- and pathway analysis (for example, see, Ramanan et al., 2012; Carter et al., 2013). The former is based on the network concept derived from mathematical graph theory and typically represents a biological component (e.g., a gene) as a node and physical-, genetic-, and/or functional interactions as a link in the network to visualize and interpret the omics data (“data-driven approach”). On the other hand, pathway analysis is a knowledge-based approach that involves the associated biochemical pathway. Enrichment analysis approaches can be combined with pathway analysis to evaluate whether a particular molecular group is significantly over-represented. Examples are gene set enrichment analysis (Hung et al., 2012), Metabolite Set Enrichment Analysis (MSEA) (Xia and Wishart, 2010), and other functional enrichment analyses using gene ontology (GO) and biochemical pathways (for comprehensive reviews see Chagoyen and Pazos, 2013 or Khatri et al., 2012).

For a holistic view of plant metabolisms, measuring the metabolic flux by experimental flux analysis, e.g., metabolic flux analysis (MFA) (Libourel and Shachar-Hill, 2008; Sweetlove et al., 2014) or *in silico* flux modeling, e.g., flux balance analysis (FBA) (Kruger and Ratcliffe, 2012; Junker, 2014) is also important. FBA is a constraint-based approach for predicting flux through reactions in a quantitative manner (Orth et al., 2010; Sweetlove and Ratcliffe, 2011); it complements experimental flux analysis. It does not use knowledge of kinetic parameters from metabolic reactions but relies solely on the stoichiometric balance assuming steady-state conditions. These models can be extended to a level that almost fully includes the metabolism. Indeed, the past few years have seen an increase in the use of genome-scale metabolic models in plants (Collakova et al., 2012; Seaver et al., 2012; De Oliveira Dal'molin and Nielsen, 2013). Integrated network analysis by combining omics data with mathematical models has become popular. In this review we focus on the latest cutting-edge computational advances for analyzing omics networks and performing pathway analysis. We highlight (1) network visualization tools, (2) pathway analyses, (3) genome-scale metabolic reconstruction, and (4) the integration of high-throughput experimental data with mathematical modeling. These topics correspond to interaction-based and constraint-based approaches to the mathematical modeling of cellular networks as classified by Stelling (2004), Lewis et al. (2012).

## Network visualization and pathway analysis tools for interaction-based approaches

The relationship between the biological components of a biological network includes four types of interactions: physical interactions (e.g., drug targets Yildirim et al., 2007 and protein-protein interactions Brandao et al., 2009), genetic interactions (Costanzo et al., 2010), and functional interactions (e.g., biochemical/signaling pathways Caspi et al., 2012; Kanehisa et al., 2014). Interaction-based approaches such as topological analysis (e.g., shortest path search Yu et al., 2014, centrality analysis Carrera et al., 2009, and network module detection Altaf-Ul-Amin et al., 2006), correlation network analysis (Provart, 2012), or enrichment analysis (Hung et al., 2012) have been used to construct and analyze biological networks from omics data. For example, GeneMANIA (Montojo et al., 2010; Zuberi et al., 2013) is a web-based interaction network for the visualization of physical, genetic, and functional interactions. Network visualization tools (e.g., igraph, http://igraph.org/) can not only describe a biological network, but also calculate and perform computational analysis (for a comprehensive review see Gehlenborg et al., 2010). Furthermore, network visualization tools assist the database client and facilitate data integration (Table 1).

**Table 1:** **List of software discussed in this review: Network tools for metabolic system biology analysis and related data formats**.

**Software name**	**Description**	**Supported data formats (built-in annotation data)**	**Interface/ Language**	**Reference/Author name**	**URL**
**NETWORK AND PATHWAY ANALYSIS TOOL**					
igraph	Library for the analysis of networks. It supports topological- and centrality analysis.	(in most cases) adjacency list, edge list, GraphML	R, Python, C/C++		http://igraph.org/index.html
Networkx	A Python language software library for the analysis of networks. This package supports topological- and centrality analysis.	(in most cases) adjacency list, edge list, GraphML, json	Python		http://networkx.github.io/
CentiScaPe	Centralities analysis plug-in for Cytoscape.		Java	Scardoni et al. (2014)	http://apps.cytoscape.org/apps/centiscape
CentiLib	Centralities analysis plug-in for VANTED.		Java	Gräßler et al. (2012)	http://centilib.ipk-gatersleben.de/
BiNGO	Gene ontology enrichment analysis plug-in for Cytoscape.		Java	Maere et al. (2005)	http://apps.cytoscape.org/apps/bingo
FluxMap	FBA plug-in for VANTED.		Java	Rohn et al. (2012a)	http://vanted.ipk-gatersleben.de/addons/fluxmap/
Enrichment Map	A Cytoscape app/plug-in to perform and visualize pathway/gene set enrichment analysis.		Java	Merico et al. (2010)	http://apps.cytoscape.org/apps/enrichmentmap
**Network visualization**					
Cytoscape	A widely used biological network analysis platform. This software supports many biological network data formats and its visual appearance is fully customizable. The 'NetworkAnalyzer' default plug-in computes basic properties of the whole network. A huge number of plug-ins is available from the Cytoscape App Store. In contrast to VANTED, this software does not support KGML by default.	SBML, BioPAX, edge list, PSI-MI	Java	Smoot et al. (2011)	http://www.cytoscape.org/
KEGGscape	A Cytoscape plug-in for KGML import.	KGML	Java	Nishida et al. (2014)	http://apps.cytoscape.org/apps/keggscape
VANTED	Along with Cytoscape, a popular network analysis software that also supports many biological network data formats. Its visual appearance is fully customizable.	SBML, BioPAX, edge list, KGML, DOT	Java	Rohn et al. (2012b)	http://VANTED.ipk-gatersleben.de/
Kappa-View	A web-based correlation network viewer on metabolic pathway maps.	User's own omics data (AraCyc version8)	Java	Sakurai et al. (2011)	http://kpv.kazusa.or.jp/
MapMan	The view is not a network graph, but it is useful for displaying omics data onto diagrams of metabolic pathways or other processes.	User's own omics data (MapMan ontology)	Java	Usadel et al. (2005)	http://mapman.gabipd.org/
Pathview	An R/Bioconductor package for pathway-based data integration and visualization.	Bioconductor compliant data	R	Luo and Brouwer (2013)	http://www.bioconductor.org/packages/release/bioc/html/pathview.html
GeneMANIA	A web-based interaction network (including physical and genetic interactions, and co-expression and prediction networks) viewer. Also accessible via a Cytoscape plug-in.	Gene list	Java	Montojo et al. (2014)	http://genemania.org/
CellDesigner	A software for browsing and modifying existing SBML models.	SBML	Java	Funahashi et al. (2008)	http://www.celldesigner.org/
PathVisio	Pathway drawing and analysis tool for WikiPathways (http://www.wikipathways.org).	GPML	Java	Van Iersel et al. (2008)	http://www.pathvisio.org/
**Data format**					
KGML	XML for the KEGG PATHWAY database (available without the KEGG FTP academic subscription). Commonly used to reconstruct a KEGG pathway network layout. It can be used for the visualization of pathway maps of a multitude of different organisms.			Kanehisa et al. (2014)	http://www.kegg.jp/kegg/xml/
BioPAX	General format for pathway data. It is defined in OWL and represented in the RDF/XML format. Its main focus is data exchange and integration.			Demir et al. (2010)	http://www.biopax.org/
SBML	Tuned and commonly used format for mathematical models of biological networks/pathways. Almost all metabolic reconstructions are written in this format.			Hucka et al. (2003)	http://sbml.org/
PSI-MI	Suitable format for representing details about molecular-, especially protein-protein interaction data. Arabidopsis Interactome Mapping Consortium data are distributed in this format.			Van Roey et al. (2013)	https://code.google.com/p/psimi/

### Network visualization and pathway analysis tools

Data analysis of biological networks by graph representations includes topological analysis (for an example see, Toubiana et al., 2013). For functional networks, correlation and enrichment analyses can be used. Correlation analysis is based on associations between biological components (e.g., genes and metabolites). The Pearson correlation coefficient is a special case of association that evaluates linear relationships among molecular abundances (Kusano and Fukushima, 2013). Enrichment analysis uses a given molecular group such as gene ontology and biochemical pathways. Some network visualization tools implement these approaches while others involve independent, plug-in software modules (e.g., Cytoscape Smoot et al., 2011 and VANTED Rohn et al., 2012b). Cytoscape apps/plug-ins include BiNGO (Maere et al., 2005) for GO enrichment analysis and FluxMap for FBA (Rohn et al., 2012a). Network analysis platforms such as Enrichment map (Merico et al., 2010) feature system flexibility and expandability for omics data. Most network visualization tools manage and visualize network data that correspond to the type of interaction. For example, when performing a quality check of protein-protein interaction data generated from a high-throughput yeast two-hybrid screening system, these tools can visualize a giant network component from a large number of interactions (for example see Arabidopsis Interactome Mapping Consortium, 2011). For functional interactions, when mapping transcriptomics profiles onto metabolic pathways, a pathway-level representation of the gene expressions involved can be assessed (Usadel et al., 2005; Sakurai et al., 2011). The network visualization tool requires a sophisticated function, structured and controlled functional categories, and vocabularies, to inspect the profile data on a pathway. Two typical functional categories are the Kyoto Encyclopedia of Genes and Genomes (KEGG) (Kanehisa et al., 2014) and GO, which can be used to evaluate perturbed pathways in the omics data. For physical interaction networks, concerted efforts are being made to share data formats that visualize biological networks such as the PSI-MI format (Van Roey et al., 2013) in IntAct (Kerrien et al., 2012).

### Network/pathway data formats

There are many data formats for functional interaction networks, especially biochemical pathway databases. As KEGG XML (KGML) (Kanehisa et al., 2014), BioPAX (Demir et al., 2010), and SBML (Hucka et al., 2003) are available for pathway data exchanges, a network visualization tool that implements and supports these data formats as import and export functions is desirable. For example, AraCyc (Zhang et al., 2005) and Arabidopsis Reactome (Tsesmetzis et al., 2008) are also represented in the BioPAX format (Table 1). BioPAX is defined in Web Ontology Language (OWL); it contains the most comprehensive ontology for representing pathway knowledge. It can also serve as a Resource Description Framework (RDF) for describing information on the world-wide-web (Jupp et al., 2014) and it is expected to utilize semantic data integration. According to Strömbäck and Lambrix (2005), SBML (Hucka et al., 2003) is the most widely used and finely tuned format for mathematical models (e.g., the FBA model). KEGG pathways are manually drawn and the layout is created by domain experts. Because KGML includes all KEGG pathway layout information, it uses another SBML-based software/database to reconstruct a pathway map. Although it is a *de facto* standard network visualization tool and supports most data formats, Cytoscape (Smoot et al., 2011) cannot seamlessly integrate all data resources irrespective of the data schema and controlled ontology. There are considerable community-wide efforts in the sustainable development and integration of various database resources with RDF (for example see BioHackathon Katayama et al., 2014, the Rhea database Alcantara et al., 2012, and Path2Models Buchel et al., 2013). The number of Wiki-based databases (Arita, 2009) is also increasing; this community curation process includes WikiPathways (Hanumappa et al., 2013) with PathVisio (Van Iersel et al., 2008) and LipidBank (http://jcbl.jp/wiki/Category:LB). Currently, several pathway resources are often combined with SBML to use FBA (see Section Genome-scale metabolic reconstruction in plants and constraint-based approaches).

### Visualization of omics data for exploring biological networks

**Figure 1:**
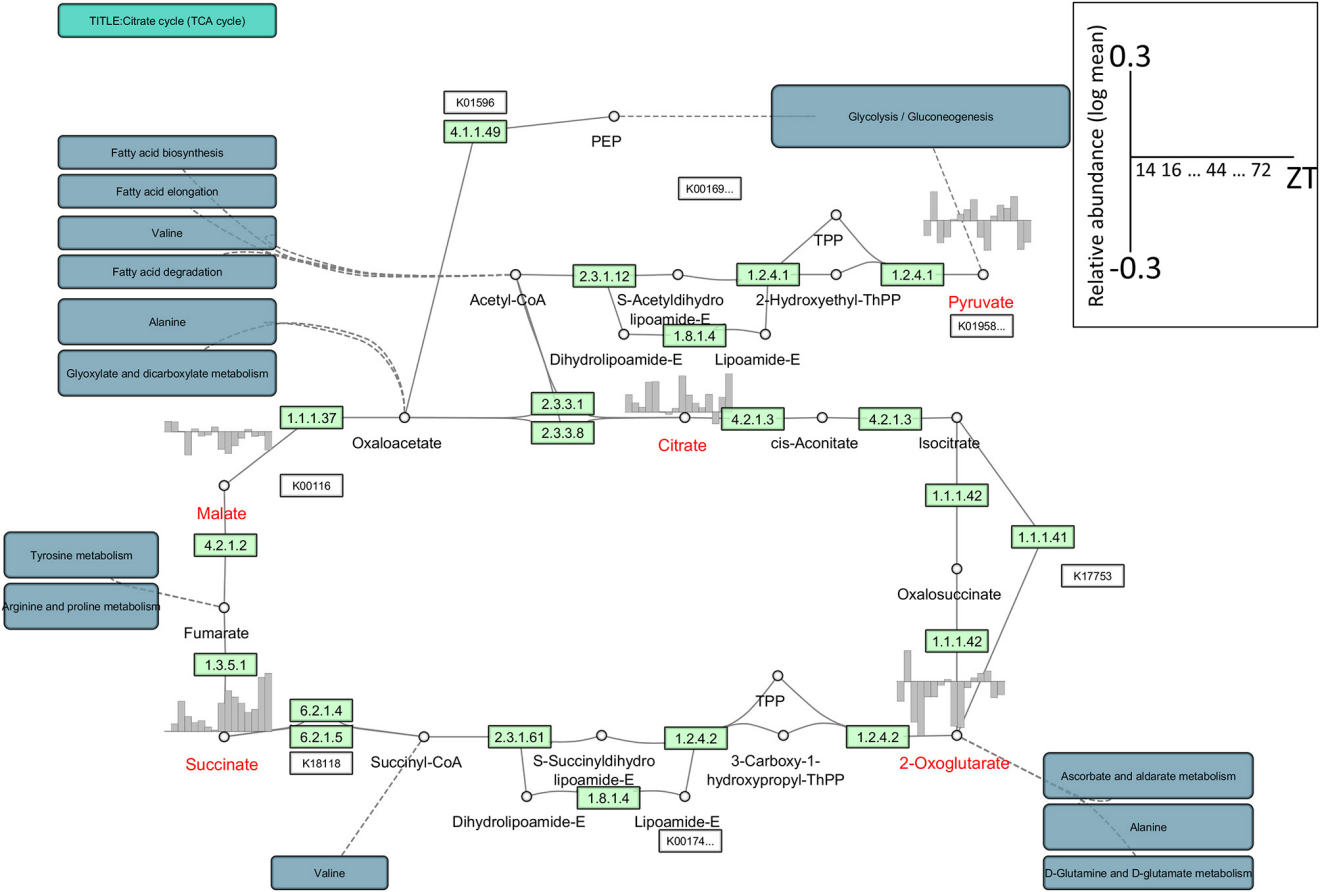
**An example of the network representation of time-series metabolome data in Arabidopsis using KEGGscape (http://apps.cytoscape.org/apps/keggscape)**. The datasets were sampled with 4-h resolution under a 16 h/8 h light/dark cycle at 20°C (Espinoza et al., 2010). We used the KEGG pathway map (ath00020), the tricarboxylic acid (TCA) cycle, or the citrate cycle. We queried MetMask (Redestig et al., 2010) for a list of KEGG compound IDs associated with a list of predefined metabolite names and picked up the most pathway-mapped KEGG compound ID for each metabolite. Metabolite names shown in red represent detected compounds in the dataset. The diurnal changes were visualized on bar charts ranging from −0.3 to 0.3 in log-mean values. ZT, Zeitgeber time.

Optimal network visualization tools must allow the seamless integration of multiple data resources and their comparison, irrespective of differences in the data formats generated by primary data providers. However, with currently available network visualization tools, the integration of different data resources remains difficult although the visualization of omics data has been partially achieved with tools such as VANTED and Pathview (Luo and Brouwer, 2013). As an example, to visualize metabolomic data we used Cytoscape with its app/plug-in KEGGscape (http://apps.cytoscape.org/apps/keggscape) (Nishida et al., 2014) and VANTED. KEGGscape supports KEGG pathway files in KGML format and reproduces KEGG pathway diagrams as a standard network object in Cytoscape. Users can easily integrate their own datasets with biologist-friendly KEGG pathway diagrams. Figure 1 is a network representation of the time-series metabolome (Espinoza et al., 2010) in *Arabidopsis thaliana* using KEGGscape. We integrated the metabolite profiles and the tricarboxylic acid (TCA) cycle with the KEGG compound IDs as the keys. Although Cytoscape and VANTED are different in design, both tools can visualize the same figure (Supplemental Figure S1) and we posit that they will be widely used to visualize omics-profiles on pathway maps. Such network visualization allows users to consider pathway-related profile variations that cannot be inferred immediately from the profile.

## Genome-scale metabolic reconstruction in plants and constraint-based approaches

Both MFA and FBA use stoichiometric simulation to estimate and predict cellular metabolic flux. Although MFA with ^13^C labeling is the most promising approach to characterize metabolic phenotypes in a cell, technological issues prevent its application to complete metabolisms. In this section we focus on genome-scale metabolic reconstruction and FBA.

### A genome-scale metabolic reconstruction

Due to the already extremely large and growing amount of genomic sequences yielded by high-throughput techniques, metabolism reconstruction from an organism's genome sequence has become possible (Thiele and Palsson, 2010). Although the metabolism has been reconstructed for only a few of the sequenced plant genomes, it has been modeled in some plants and crops (Seaver et al., 2012; De Oliveira Dal'molin and Nielsen, 2013). The first step in metabolic reconstruction from genome sequences involves the collection and integration of compounds, enzymes, genes, and curated published pathway databases. Subsequently, gene-protein-reaction (GPR) relationships in an organism (Fell et al., 2010) are identified and a stoichiometric matrix consisting of substances and reactions is generated. This draft metabolism requires further curation including metabolic gap filling and FBA. To explore flux states computationally, FBA uses the optimization of an objective function and predicts the growth rate of an organism or the production rate of industrially and medicinally important metabolites (Feist and Palsson, 2010). The collection of information on the biomass including proteins, amino acids, and lipid(s) as an objective function is required. Because these steps tend to be time-consuming, rapid algorithms for reconstructing genome-scale metabolisms have been developed (Chen et al., 2012; Kim et al., 2012). On SEED (Henry et al., 2010) and PlantSEED (Seaver et al., 2014) servers a significant number of genome-scale metabolisms in different organisms has already been reconstructed. In this review we do not present a comprehensive review of software tools/algorithms involved in reconstructing a genome-scale model and FBA.

### Reconstructed plant metabolisms

The first genome-scale models in plants were designed and published for barley seeds and heterotrophic Arabidopsis cells. Grafahrend-Belau et al. (2009) constructed a compartmentalized barley seed metabolism model and performed mathematical simulations to investigate storage patterns that included responses to environmental and genetic perturbations (Grafahrend-Belau et al., 2009). Their comparison of published data for grain yields and growth rates with *in silico* data showed good reproducibility, indicating the usefulness of their model for predicting the seed storage metabolism. Poolman et al. (2009) generated an Arabidopsis genome-scale metabolic model using the AraCyc database (Poolman et al., 2009). They demonstrated that only 15% of the reactions in the reconstructed network (“minimal network”) were required to produce amino acids, nucleotides, and other biomass components. For the Arabidopsis metabolism, two other models are available, i.e., AraGEM (De Oliveira Dal'molin et al., 2010) and the model of Radrich (Radrich et al., 2010). In addition, 7 tissue-specific models for Arabidopsis have been presented (Mintz-Oron et al., 2012). The model of Poolman et al. (2009) was extended and updated to include more information on the subcellular localization of enzymes and transport reactions (Cheung et al., 2013) and to model the leaf metabolism over a day-night diel cycle (Cheung et al., 2014). The approach with MFA demonstrated a marked improvement in the quantitative match between predicted- and experimentally-estimated fluxes. To assess the central carbon partitioning and enzyme costs precisely, Arnold and Nikoloski (2014) newly reconstructed the Arabidopsis metabolism based on genomic and bibliomic data that included biochemical, genomic, and genetic information on compartmentalization and transport processes. Their model produced all amino acids and was able to estimate various cell performances (Arnold and Nikoloski, 2014). De Oliveira Dal'molin et al. (2010) constructed a genome-scale metabolic model for C4 plants (C4GEM) (Dal'molin et al., 2010), Saha et al. (2011) modeled the maize metabolism that contains maize-specific GPR (Saha et al., 2011), and Grafahrend-Belau et al. (2013) developed multi-scale metabolic modeling (MMM) for predicting the plant metabolism at the whole plant level; their barley model has provided significant insights into the metabolic capacity for yield stability and crop improvement (Grafahrend-Belau et al., 2013).

## Integration of high-throughput experimental data with mathematical modeling

The integration of omics data and mathematical models is a promising approach to gain a better understanding of plant metabolisms (Bordbar et al., 2014; Saha et al., 2014). Integrated concepts involving FBA make it possible to predict genotype-phenotype relationships and to gain important insights into the metabolic network capacity of an organism (Blazier and Papin, 2012). For example, an integrated model in which gene expression was combined with a metabolic network (ME model) in *Escherichia coli* increased the accuracy for predicting feasible and computable phenotypes that respond to optimal growth conditions (Lerman et al., 2012). Karr et al. (2012) showed that a whole-cell model in *Mycoplasma genitalium* was useful for describing protein-DNA binding and correlations between DNA replication and its initiation. Their findings indicate that the integrated approach makes it possible to study previously unknown biological processes in a cell. These earlier studies demonstrated that high-throughput omics data are available as a constraint parameter for generating high-quality metabolic models. The model-building algorithm (MBA) developed by Jerby et al. (2010) is used to construct tissue-specific metabolisms from generic models and omics data (Jerby et al., 2010). Gene Inactivity Moderated by Metabolism and Expression (GIMME) (Becker and Palsson, 2008) is based on the premise that gene expression data correlate with metabolic fluxes and the user's pre-defined threshold of expression levels; GIMME removes reactions with expression levels lower than the threshold from the model and evaluates metabolic capacities. iMAT (Folger et al., 2011) is similar to GIMME; it is based on the discretization of input expression data and returns predictive optimal flux with confidence values over all network reactions. Metabolic Adjustment by Differential Expression (MADE) (Jensen and Papin, 2011) uses significant changes in transcript levels between two or more conditions classified into so-called “switch” approaches. This is then used to identify on/off reaction fluxes based on threshold expression levels in the constraint-based models (Hyduke et al., 2013; Saha et al., 2014).

Two other approaches exist, they are known as “valve” approaches and they allow the use of gene expression data to limit the maximum activity of an enzyme. The first, E-FLUX (Colijn et al., 2009), uses maximum flux constraints as a function of measured transcript levels without binalization of the expression data. The other approach is GIM3E, it does not apply arbitrary cutoffs for expression levels (Schmidt et al., 2013). Protein data can also be included. PROM (Chandrasekaran and Price, 2010) invokes a threshold to determine whether an enzyme is in its active or inactive state and uses information about regulatory interactions including transcription factor-target gene interactions. Integrative Omics-Metabolic Analysis (IOMA) (Yizhak et al., 2010) integrates proteomic and metabolomic data into a genome-scale metabolic model by evaluating kinetic rate equations subject to quantitative omics measurements. Machado and Herrgard (2014) who systematically evaluated different methods for the integration of transcriptome data into constraint-based models reported that no robust approaches worked well under all examinations (Machado and Herrgard, 2014). In plant science, Topfer et al. (2013) performed E-FLUX on the Arabidopsis genome-scale models created by Mintz-Oron et al. (2012) and used high-resolution time-series transcriptome data (Caldana et al., 2011) to investigate metabolic capacities in response to different environmental changes (Topfer et al., 2013). Their optimization-based approach was able to characterize many aspects of the metabolic behaviors and functions in response to a changing environment. In an attempt to integrate metabolome data with constraint-based mathematical models, Nagele and Weckwerth (2013) developed a complementary approach to obtain a comprehensive view of metabolic capacities in Arabidopsis leaves (Nagele and Weckwerth, 2013). Using experimentally accessible metabolites and the Mintz-Oron model (Mintz-Oron et al., 2012) they derived a metabolic model that yielded an overview of metabolic phenotypes perturbed by genetic and environmental differences.

## Future perspectives

Metabolic network models have contributed to the study of metabolic capacity in response to environmental and genetic perturbations and to the identification of feasible metabolic networks in an organism. They provided important clues about genotype-phenotype relationships. Reconstruction of the metabolism from genome sequences is a non-trivial task that requires not only effective computational tools but also integrated knowledge-based systems. For comprehensive reconstructions, improved technologies, including more sophisticated algorithms and tools, better software frameworks for multiple omics data analyses, improved visualization of biological networks, and more effective integration of data with mathematical models are needed. Multi-omics data that include the genome, transcriptome, proteome, and metabolome plus mathematical modeling can be expected to deepen our knowledge of complex plant metabolisms and to illuminate unexplored biological processes.

### Conflict of interest statement

The authors declare that the research was conducted in the absence of any commercial or financial relationships that could be construed as a potential conflict of interest.
