# Supplementary figures and images for: Integrated network analysis and effective tools in plant systems biology
Source: Front Plant Sci. 2014 Nov 4;5:598. doi: 10.3389/fpls.2014.00598 (PMC4219401; doi:10.3389/fpls.2014.00598)

## Slide 1
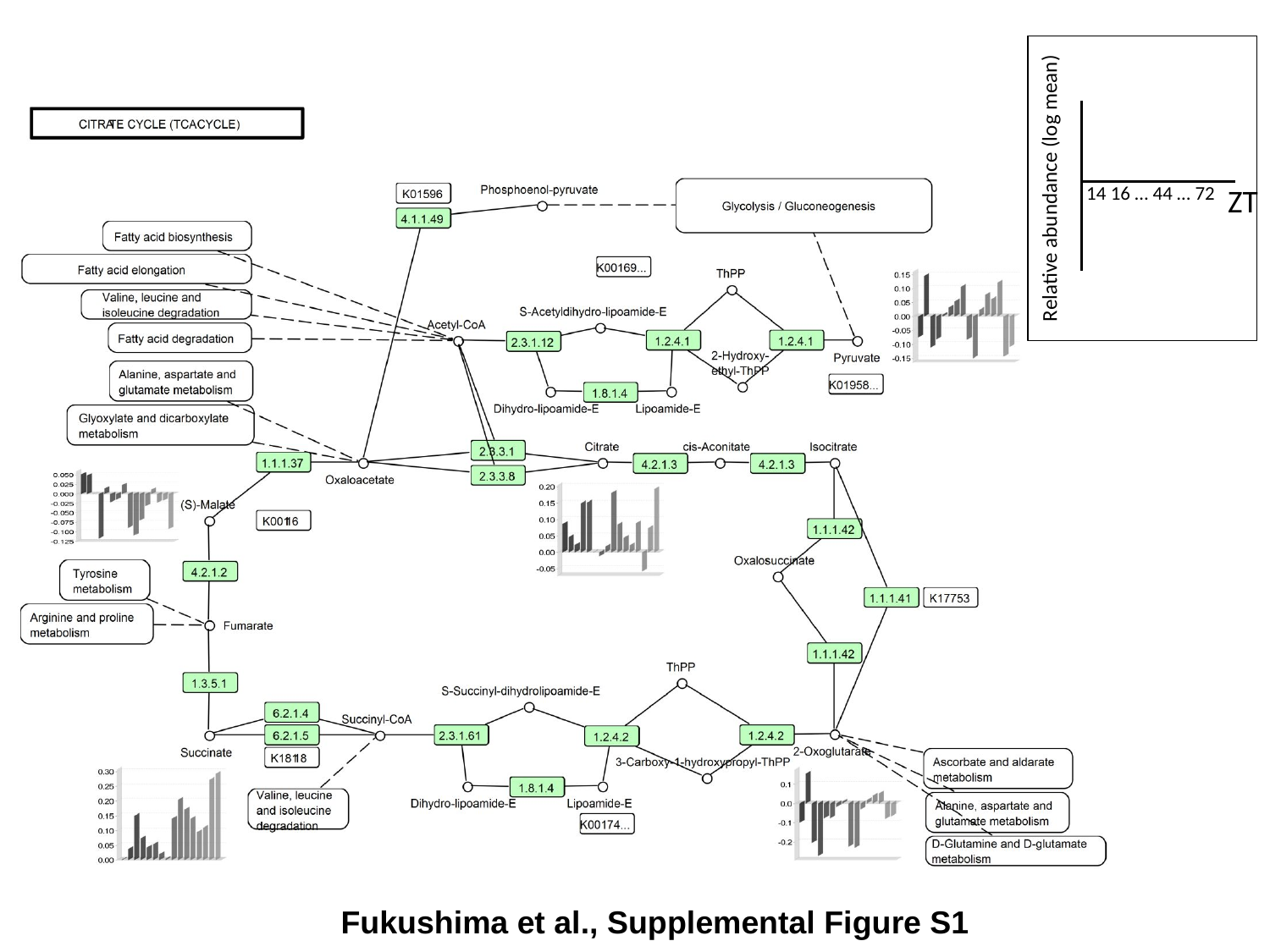

Relative abundance (log mean)
14 16 … 44 … 72
ZT
Fukushima et al., Supplemental Figure S1

Supplement: Supplementary file 1 [file Presentation1.PPTX]
